# Methane Inhalation Protects Against Lung Ischemia-Reperfusion Injury in Rats by Regulating Pulmonary Surfactant *via* the Nrf2 Pathway

**DOI:** 10.3389/fphys.2021.615974

**Published:** 2021-05-12

**Authors:** Bing Zhang, Xiaojun Tian, Guangqi Li, Han Zhao, Xuan Wang, Yanwei Yin, Junmin Yu, Chao Meng

**Affiliations:** ^1^Department of Anesthesiology, The Affiliated Hospital of Qingdao University, Qingdao, China; ^2^Department of Radiology, The Affiliated Hospital of Qingdao University, Qingdao, China; ^3^Department of Pathology, The Affiliated Hospital of Qingdao University, Qingdao, China; ^4^Department of Pain Management, The Affiliated Hospital of Qingdao University, Qingdao, China

**Keywords:** methane, lung ischemia-reperfusion injury, pulmonary surfactant, lung function, Nrf2

## Abstract

Methane (CH_4_) exerted protective effects against lung ischemia-reperfusion (I/R) injury, but the mechanism remains unclear, especially the role of pulmonary surfactant. Therefore, this study aimed to explore the effects of CH_4_ inhalation on pulmonary surfactant in rat lung I/R injury and to elucidate the mechanism. Rats were randomly divided into three groups (*n* = 6): the sham, I/R control, and I/R CH_4_ groups. In the sham group, only thoracotomy was performed on the rats. In the I/R control and I/R CH_4_ groups, the rats underwent left hilum occlusion for 90 min, followed by reperfusion for 180 min and ventilation with O_2_ or 2.5% CH_4_, respectively. Compared with those of the sham group, the levels of large surfactant aggregates (LAs) in pulmonary surfactant, lung compliance, oxygenation decreased, the small surfactant aggregates (SAs), inflammatory response, oxidative stress injury, and cell apoptosis increased in the control group (*P* < 0.05). Compared to the control treatment, CH_4_ increased LA (0.42 ± 0.06 vs. 0.31 ± 0.09 mg/kg), oxygenation (201 ± 11 vs. 151 ± 14 mmHg), and lung compliance (16.8 ± 1.0 vs. 11.5 ± 1.3 ml/kg), as well as total antioxidant capacity and Nrf2 protein expression and decreased the inflammatory response and number of apoptotic cells (*P* < 0.05). In conclusion, CH_4_ inhalation decreased oxidative stress injury, inflammatory response, and cell apoptosis, and improved lung function through Nrf2-mediated pulmonary surfactant regulation in rat lung I/R injury.

## Introduction

Lung ischemia-reperfusion (I/R) injury can occur during many clinical events, such as lung transplantation, cardiopulmonary bypass, pulmonary embolism, single-lung ventilation, resuscitation for circulatory arrest, and shock, and can contribute to severe organ failure and increased mortality in patients (Tejwani et al., 2016; Saito et al., 2019). Lung I/R injury is a complex pathological process that includes oxidative stress injury, the inflammation response, and cell apoptosis and is characterized by poor oxygenation, tissue edema, and neutrophil infiltration (den Hengst et al., 2010). Many measures have been applied, such as ischemic preconditioning, protective drug injection, gene therapy, and protective gas inhalation (Meng et al., 2016a; Jiang et al., 2019). Unfortunately, these methods still cannot effectively control lung I/R injury. Thus, a novel protective strategy needs to be explored.

Oxidative stress injury is an important factor contributing to lung I/R injury, leading to the intracellular generation of reactive oxygen species (ROS) (Boros and Keppler, 2019). The accelerated generation of ROS is a major cause of cell apoptosis and inflammation and exacerbates lung injury during the ischemia and reperfusion phase (Yang et al., 2019). Nuclear factor E2-related factor 2 (Nrf2), a redox-sensitive transcription factor, is commonly believed to play a key role in antioxidant processes (Meng et al., 2017). In our previous study, we found that Nrf2 exerted antioxidant effects against lung I/R injury in a rat lung transplantation model (Meng et al., 2017). Thus, the Nrf2 pathway may be a crucial mechanism in lung I/R injury.

Pulmonary surfactant consists of phospholipids and surfactant proteins (Yang et al., 2020). Pulmonary surfactant can stabilize the alveoli at the end of exhalation and prevent collapse by maintaining alveolar surface tension (Topercerova et al., 2019). Pulmonary surfactant can regulate pulmonary inflammation and cell apoptosis via IL-8(Yang et al., 2020), and is affected by oxygen free radicals (Mathew and Sarada, 2018). Thus, pulmonary surfactant may play an important role in lung I/R injury. In addition, a study found that the Nrf2 pathway was closely related to changes in pulmonary surfactant expression in A549 cells (Mathew and Sarada, 2018). Thus, during lung I/R injury, Nrf2-mediated pulmonary surfactant regulation may play a role.

Methane (CH_4_), the simplest alkane and the most abundant organic gas in the atmosphere, is commonly used as a type of fuel (Liu et al., 2012). Although CH_4_ is widely considered a biologically inactive gas, several researchers have focused on to its biological characteristics, and several studies have demonstrated the therapeutic effects of CH_4_ in many organs associated with I/R injury via anti-oxidant, anti-inflammatory, and anti-apoptotic properties (Li et al., 2017). However, the effects of CH_4_ on pulmonary surfactant and the specific mechanism remain unclear. Therefore, this study aimed to observe the effects of CH_4_ inhalation on pulmonary surfactant and lung injury induced by I/R in rats and to explore the potential role of the Nrf2 pathway.

## Materials and Methods

### Animals

Adult male Wistar rats weighing 250–280 g were purchased from the Experiment Center of the Affiliated Hospital of Qingdao University. The animals were housed at cage feeding racks in a temperature-controlled room with free access to a standard diet and water for 1 week before the experiment. The Ethics Committee of the Affiliated Hospital of Qingdao University approved all experiments and procedures in this study (No. AHQU20180702).

### Lung I/R Injury Model

The rats were anesthetized by an intraperitoneal injection of sodium pentobarbital (60 mg/kg) and intubated with a tracheal cannula. Then, the rats were ventilated with a tidal volume of 8 ml/kg at a respiratory rate of 50 breaths/min. The femoral artery and vein were cannulated for blood pressure monitoring and drug administration, respectively. After a left lateral thoracotomy, the left lung hilum was clamped with a non-clash microclip at the end of expiration 5 min after the administration of heparin (50 IU/animal). The tidal volume was reduced to 6 ml/kg during clamping. After a 90-min ischemic period, the microclip was removed for 180 min of reperfusion. Then, the tidal volume was adjusted to 8 ml/kg. During the experiment, sodium pentobarbital and rocuronium bromide were used to maintain anesthesia and muscle relaxation. The breath rate was adjusted to maintain an arterial carbon dioxide tension (PaCO_2_) of 35–45 mmHg, and the body temperature was maintained between 37.5 and 38.5°C by a heating blanket.

### Experimental Protocol

**FIGURE 1:**
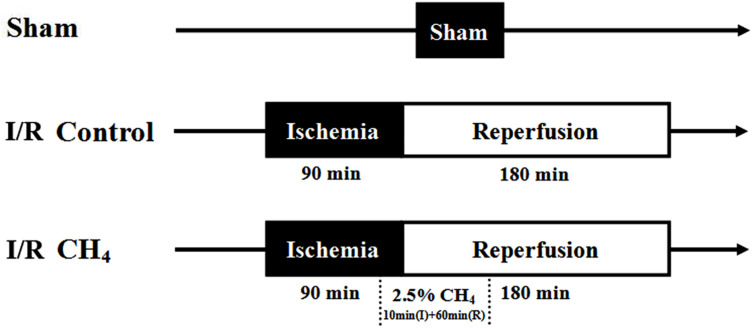
Study design. I, ischemia; R, reperfusion.

The rats were randomly divided into three groups (*n* = 6): the sham group, the I/R control group, and the I/R CH_4_ group. In the sham group, the rats underwent thoracotomy and were ventilated with 40% O_2_ + 60% N_2_ without ischemia and reperfusion. The rats in the I/R control group underwent I/R and were ventilated with 40% O_2_ + 60% N_2_. The rats in the I/R CH_4_ group underwent I/R and were ventilated with 2.5% CH_4_ + 40% O_2_ + 57.5% N_2_ beginning 10 min before the end of the 90-min ischemic period until 60 min after the beginning of the 180-min reperfusion period. Then the rats in the I/R CH_4_ group were ventilated with 40% O_2_ + 60% N_2_ (Figure 1).

### Blood Gas Analysis

Arterial blood gas analysis was performed at baseline (3 min after ventilation), at 60 min after ischemia, and at 60, 120, and 180 min after reperfusion and were recorded as T0–T4, respectively. At the end of the experiment, blood from the left pulmonary vein was collected for blood gas analysis (Rapid Lab 248, Bayer, Medfield, United States).

### Determination of the Lung Static Pressure-Volume (P-V) Curve

After 180 min of reperfusion, the rats were euthanized via exsanguination. Median sternotomies were performed immediately, and both right and left lungs were isolated with a tracheal cannula. Then, the right lung hilum was ligated. The tracheal cannula was connected to an apparatus to measure the static P-V curve of the left lung. Airway pressure was increased to 30 cm H_2_O before being decreased to 0 cm H_2_O in stepwise intervals of 5 cm. After 1 min of stabilization, the lung volume was recorded through gas compression in the apparatus (Meng et al., 2016b).

### Detection of Pulmonary Surfactant

After the P-V curve determination, the left lung was infused with 10 ml of cold normal saline three times. Each wash was infused slowly, withdrawn gently, and re-infused after 1 min of stabilization three times (Li et al., 2018). The cumulative volume of bronchoalveolar lavage fluid (BALF) was collected, and 1 ml of BALF was used to count the number of total cells and neutrophils. The rest of the BALF was centrifuged at 150 g for 10 min, and the supernatant was collected to measure phospholipids. The remaining sample was centrifuged at 40,000 g for 15 min to measure the large surfactant aggregates (LAs) and small surfactant aggregates (SAs). The phosphorus measurement was used to determine the level of phospholipids using a phosphate assay kit (Jiancheng Bio-Technology, Nanjing, China).

### Detection of Inflammatory Indices and Oxidative Stress Parameters

After 180 min of reperfusion, the lower lobe of the left lung was homogenized with cold normal saline to determine myeloperoxidase (MPO) activity using a regent kit (Beyotime Biotechnology, Shanghai, China). The supernatants were collected to measure the interleukin (IL)-10, IL-1β, and tumor necrosis factor (TNF)-α by enzyme-linked immunosorbent assay kits (R&D Systems, Minneapolis, MN, United States), and the malondialdehyde (MDA) levels, superoxide dismutase (SOD) activity, and total antioxidant capacity (T-AOC) were measured by commercially available kits and a BCA kit (Beyotime Biotechnology, Shanghai, China). The BCA kit was used for total protein detection, which was used to calculate MDA, SOD, and T-AOC. The upper lobe of the left lung was dried at 80°C for 72 h to a constant weight to determine the wet-to-dry weight (W/D) ratio.

### Detection of Histopathology and Cell Apoptosis

The middle lobe of the left lung was used for hematoxylin-eosin (H&E) staining and terminal deoxynucleotidyl transferase dUTP nick end-labeling (TUNEL) staining (Zhongshan Golden Bridge Biotechnology, Beijing, China). The lung injury score (LIS) (Meng et al., 2016a) was evaluated by histopathology based on the following criteria: (1) neutrophil infiltration, (2) airway epithelial cell damage, (3) interstitial edema, (4) hyaline membrane formation, and (5) hemorrhage. Each criteria have five scores as follows: normal = 0, minimal change = 1, mild change = 2, moderate change = 3, and severe change = 4. The apoptotic index (AI), a measure of the number of positive cells per 100 cells in five random fields of the same section, was evaluated by TUNEL staining (Meng et al., 2017). All sections were examined by a pathologist who was blinded to the study conditions.

### Western Blot Analysis

The lower lobe of the left lung was homogenized in lysis buffer, and the proteins were collected. For the detection of Nrf2 and NF-κB, the cytoplasmic component and nuclear component were isolated by treating a nuclear protein extraction kit (Beyotime Biotechnology, Shanghai, China) and centrifuged at 12,000 g for 10 min at 4°C. After the protein concentration was measured by a BCA kit, 50-μg protein samples were separated by SDS-PAGE and transferred onto a PVDF membrane. The membrane was blocked in 5% non-fat dry milk, followed by incubation with pro-caspase-3 (1:8,000, Abcam, United States), cleaved-caspase-3 (1:500, Abcam, United States), pro-caspase-9 (1:800, Abcam, United States), cleaved-caspase-9 (1:1,000, Abcam, United States), Nrf2 (1:5,000, Abcam, United States), and NF-κB (1:3,000, Abcam, United States) primary antibodies overnight at 4°C. After being washed and incubated with secondary antibody (1:5,000, Zhongshan Golden Bridge Biotechnology, Beijing, China) at room temperature for 1 h, the proteins were visualized with the enhanced chemiluminescence reagent (GE Healthcare Bio-Sciences, Pittsburgh, PA, United States), analyzed using ImageJ version 1.61 software (National Institutes of Health, Bethesda, MD, United States) and normalized to β-actin and Lamin B.

### Statistical Analysis

All quantitative data are expressed as the mean values ± standard deviations. Differences between groups were assessed by one-way analysis of variance (ANOVA) followed by the Student-Newman-Keuls test. The repeated data were analyzed through repeated analysis of variance followed by Dunnett’s test. The non-parametric method with the Kruskal-Wallis test was used for the analysis of LIS data. A value of *P* < 0.05 was considered statistically significant. The statistical analyses were performed with SPSS 20.0 software (SPSS, Chicago, IL).

## Results

### Experiment-Related Data

All rats were operated on successfully in this study. After the microclip was removed, the chest was closed. Thus, the total time the chest was open was 91.6 ± 0.8 min in the sham group, 92.2 ± 1.2 min in the I/R control group, and 92.5 ± 1.0 min in the I/R CH_4_ group, and the differences were not statistically significant. Additionally, the mean arterial pressure (MAP) and heart rate (HR) were stable among the three groups. The baseline data in all the groups showed no significant differences.

### CH_4_ Inhalation Improved Lung Oxygenation Functions

At the T0 time point, the oxygenation index [the partial pressure of arterial oxygen (PaO_2_)/fraction of inspired oxygen (FiO_2_)] was not significantly different among the three groups. At the T1 time point, the oxygenation index was lower in the I/R control and I/R CH_4_ groups than in the sham group (*P* < 0.05). Compared with that of the sham group, the oxygenation index decreased significantly in the I/R control group and was significantly higher in the I/R CH_4_ group than in the control group at T2–T4 (*P* < 0.05). The base excess (BE) and pH values exhibited similar trends as the oxygenation index (Table 1). Additionally, there was also a similar trend in the analysis of blood from the left pulmonary vein as the relative indices shown above (Table 2).

**TABLE 1:** The indices of blood gas analysis (mean ± SD, *n = 6).*

	Group	T0	T1	T2	T3	T4
PaO_2_/FiO_2_ (mm Hg)	Sham group	241 ± 5	244 ± 6	242 ± 4	239 ± 4	245 ± 7
	I/R control group	242 ± 7	219 ± 14*	199 ± 11*	176 ± 18*	151 ± 14*
	I/R CH_4_ group	245 ± 5	222 ± 15*	217 ± 15*^#^	211 ± 13*^#^	201 ± 11*^#^
pH value	Sham group	7.39 ± 0.02	7.39 ± 0.02	7.39 ± 0.02	7.40 ± 0.01	7.40 ± 0.01
	I/R control group	7.40 ± 0.02	7.35 ± 0.03*	7.30 ± 0.03*	7.26 ± 0.01*	7.23 ± 0.03*
	I/R CH_4_ group	7.39 ± 0.01	7.35 ± 0.02*	7.33 ± 0.03*^#^	7.32 ± 0.02*^#^	7.31 ± 0.03*^#^
BE value (mmol/L)	Sham group	0.07 ± 0.02	0.08 ± 0.03	0.08 ± 0.04	0.08 ± 0.04	0.08 ± 0.03
	I/R control group	0.07 ± 0.04	–1.45 ± 0.25*	–3.07 ± 0.09*	–4.06 ± 0.30*	–4.50 ± 0.20*
	I/R CH_4_ group	0.08 ± 0.26	–1.46 ± 0.14*	–2.01 ± 0.11*^#^	–2.39 ± 0.12*^#^	–2.49 ± 0.21*^#^
PaCO_2_ (mm Hg)	Sham group	38.8 ± 1.8	38.9 ± 2.1	39.2 ± 1.8	38.9 ± 1.8	39.0 ± 1.7
	I/R control group	40.1 ± 1.2	38.1 ± 3.0	39.4 ± 2.2	38.4 ± 2.7	39.2 ± 1.6
	I/R CH_4_ group	39.2 ± 1.7	38.2 ± 1.4	39.1 ± 1.3	38.7 ± 1.0	38.1 ± 1.3

T0–T4 represented the following time points: the baseline, 60 min after ischemia, and 60, 120, and 180 min after reperfusion. PaO_2_/FiO_2_, partial pressure of arterial oxygen (PaO_2_)/fraction of inspired oxygen (FiO_2_); BE, base excess; PaCO_2_, arterial carbon dioxide tension. *P < 0.05 vs. the sham group; ^#^P < 0.05 vs. the I/R control group.

**TABLE 2:** The indices of blood gas analysis from pulmonary vein (mean ± SD, *n* = 6).

	PvO_2_/FiO_2_ (mmHg)	pH value	BE value (mmol/L)	PvCO_2_ (mm Hg)
Sham group	246 ± 9	7.40 ± 0.02	0.07 ± 0.02	38.6 ± 1.6
I/R control group	131 ± 38*	7.17 ± 0.04*	–4.27 ± 0.54*	38.9 ± 1.2
I/R CH_4_ group	188 ± 17*^#^	7.33 ± 0.04*^#^	–2.14± 0.32*^#^	38.0 ± 2.8

PvO_2_/FiO_2_, pulmonary venous oxygen tension (PvO_2_)/fraction of inspired oxygen (FiO_2_); BE, base excess. *P < 0.05 vs. the sham group;^#^*P < 0.05 vs. the I/R control group.*

### CH_4_ Inhalation Maintained Lung Tissue Structure

**FIGURE 2:**
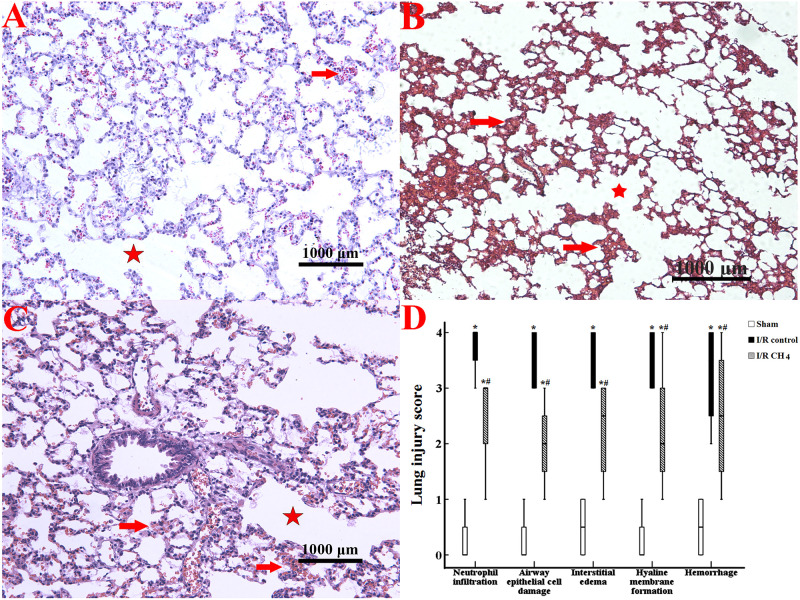
Histological analysis of lung tissues (original magnification, 40×). Paraformaldehyde-fixed sections of lung grafts were stained with hematoxylin and eosin. Lung tissues in the sham group appeared nearly normal, and the LIS for each criterion was ameliorated in the I/R CH_4_ group compared with the I/R control group. The alveolar fusion was marked by a red star, and the lung congestion was marked by a red arrow. **(A)** Sham group; **(B)** I/R control group; **(C)** I/R CH_4_ group; **(D)** lung injury score (*n* = 4). *P < 0.05 vs. the sham group; ^#^*P* < 0.05 vs. the I/R control group.

Compared with those of the sham group, more neutrophil infiltration, interstitial edema, and hemorrhage were observed in the I/R control group. Compared with those of the I/R control group, the neutrophil infiltration, interstitial edema, and hemorrhage decreased in the I/R CH_4_ group. Therefore, the neutrophil infiltration LIS in the I/R control group [3.5 (3–4)] was higher than that in the sham group [0.5 (0–1)] (*P* < 0.05) and that in the I/R CH_4_ group [2 (1–3)] was lower than that in the I/R control group (*P* < 0.05). The LISs of the other criteria also exhibited similar trends (Figure 2).

### CH_4_ Inhalation Regulated Phospholipid and Pulmonary Surfactant Proteins

**FIGURE 3:**
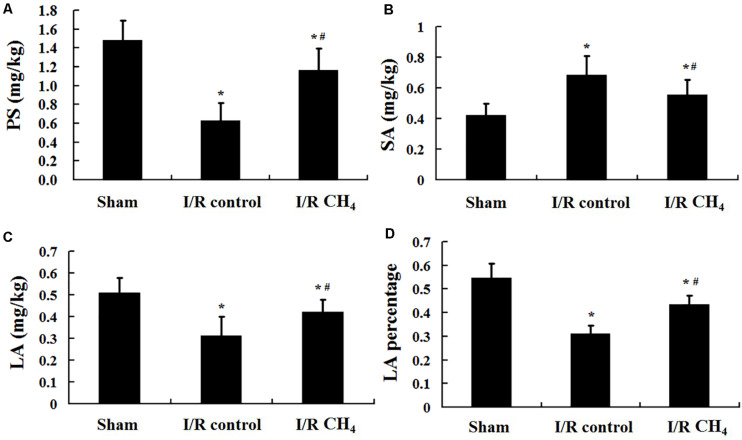
Phospholipid and pulmonary surfactant levels (*n* = 6). **(A)** content of PS; **(B)** content of SA; **(C)** content of LA; **(D)** content of LA percentage. PS: phospholipid; SA: small surfactant aggregate; LA: large surfactant aggregate. ^∗^*P* < 0.05 vs. the sham group; ^#^*P* < 0.05 vs. the I/R control group.

Compared with those of the sham group, the phospholipid level (1.48 ± 0.21 mg/kg), LA level (0.51 ± 0.07 mg/kg), and LA percentage (0.55 ± 0.06) in BALF were decreased and the SA level (0.42 ± 0.07 mg/kg) was increased in the I/R control group (0.63 ± 0.19 mg/kg, 0.31 ± 0.09 mg/kg, 0.31 ± 0.04, 0.68 ± 0.13 mg/kg) (*P* < 0.05). Compared with those of I/R control group, the phospholipid level (1.16 ± 0.24 mg/kg), LA level (0.42 ± 0.06 mg/kg), and LA percentage (0.43 ± 0.04) were increased and the SA level (0.55 ± 0.10 mg/kg) was decreased in the I/R CH_4_ group (*P* < 0.05) (Figure 3).

### CH_4_ Inhalation Improved Lung Compliance

**FIGURE 4:**
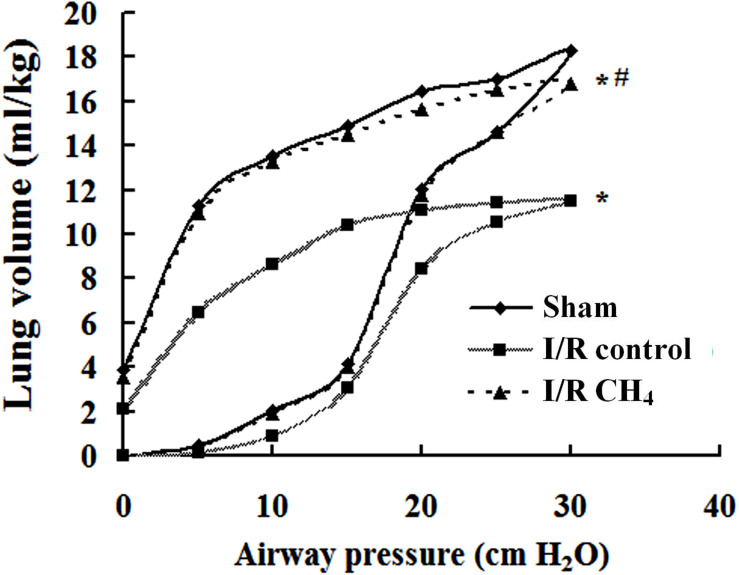
Lung static compliance (*n* = 6). Pressure-volume (P-V) curves were used to determine the static compliance of the lung. The data are presented as the mean values, and the bars are omitted for clarity. *P < 0.05 vs. the sham group; ^#^*P* < 0.05 vs. the I/R control group.

The P-V curve value of the I/R control group was significantly lower than that of the sham group, and the value of the I/R CH_4_ group was markedly higher than that of the I/R control group (*P* < 0.05). At a pressure of 30 cm H_2_O, the values in the sham, I/R control, and I/R CH_4_ groups were 18.3 ± 1.0 ml/kg, 11.5 ± 1.3 ml/kg, and 16.8 ± 1.0 ml/kg, respectively (Figure 4).

### CH_4_ Inhalation Decreased the Lung Inflammatory Response

**FIGURE 5:**
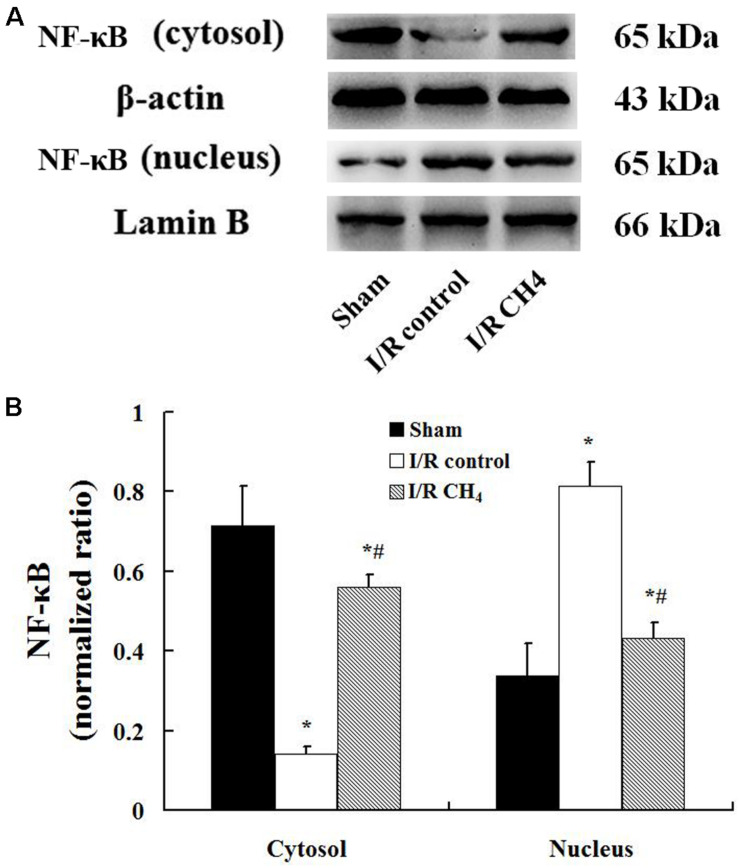
Expression of NF-κB by Western blotting (*n* = 4). The expression of NF-κB in the lung grafts was measured by Western blotting after 180 min of reperfusion in each group. **(A)** Representative bands of NF-κB, β-actin, and lamin B; **(B)** expression of NF-κB normalized to β-actin and lamin B. Data are shown as mean ± SD. NF-κB: nuclear factor kappa B. *P < 0.05 vs. the sham group; ^#^*P* < 0.05 vs. the I/R control group.

The W/D ratio in the I/R control group was significantly higher than that in the sham group, and the W/D ratio in the I/R CH_4_ group was higher than that in the I/R control group (*P* < 0.05). The total number of cells and neutrophils in BALF exhibited the same trends as the W/D ratio. Additionally, the MPO activity and the levels of IL-1β and TNF-α in the I/R control group increased significantly compared with those of the sham group, and the MPO activity and levels of IL-1β and TNF-α in the I/R CH_4_ group decreased significantly compared with those of the I/R control group (*P* < 0.05). IL-10 exhibited the opposite trend as TNF-α (Table 3). Additionally, compared with the sham group, the nucleus NF-κB protein expression in the I/R control group increased significantly, and that of the I/R CH_4_ group decreased significantly compared with the I/R control group (*P* < 0.05). NF-κB protein expression in the cytosol showed a trend that was contrary to that in the nucleus (Figure 5).

**TABLE 3:** W/D ratio and inflammatory mediators (mean ± SD, *n* = 6).

	W/D ratio	Cells in BALF (10^6^/l)	Neutrophils in BALF (%)	MPO (U/g)	IL-1β (pg/mg)	TNF-α (pg/mg)	IL-10 (pg/mg)
Sham group	3.4 ± 0.2	54.0 ± 7.9	10.0 ± 1.1	0.43 ± 0.23	98.4 ± 8.6	299.9 ± 58.5	46.8 ± 16.9
I/R control group	5.8 ± 0.7*	199.3 ± 9.8*	67.4 ± 7.5*	1.55 ± 0.38*	334.6 ± 28.0*	579.5 ± 54.5*	92.4 ± 14.1*
I/R CH_4_ group	4.4 ± 0.5*^#^	166.5 ± 9.3*^#^	46.3 ± 5.4*^#^	0.86 ± 0.22*^#^	267.7 ± 16.2*^#^	420.6 ± 48.7*^#^	140.0 ± 27.1*^#^

W/D ratio, wet weight (W)/dry weight (D) ratio; BALF, bronchoalveolar lavage fluid; MPO, myeloperoxidase; IL-1β, interleukin-1β; TNF-α, tumor necrosis factor-α. *P< 0.05 vs. the sham group;^#^*P < 0.05 vs. the I/R control group.*

### CH_4_ Inhalation Decreased Lung Oxidative Stress Injury

In the I/R control group, the MDA level was significantly higher and the SOD and T-AOC activities were significantly lower than those in the sham group (*P* < 0.05). Compared with those of the I/R control group, the MDA level decreased and the SOD and T-AOC activities increased significantly in the I/R CH_4_ group (*P* < 0.05) (Table 4).

**TABLE 4:** Oxidative stress indices (mean ± SD, *n* = 6).

	SOD (U/mg protein)	MDA (nmol/mg protein)	T-AOC (U/mg protein)
Sham group	92.8 ± 15.3	3.46 ± 0.36	2.13 ± 0.24
I/R control group	40.1 ± 8.6*	9.37 ± 1.42*	0.94 ± 0.18*
I/R CH_4_ group	74.8 ± 15.3*^#^	4.83 ± 1.21*^#^	1.51 ± 0.44*^#^

SOD, superoxide dismutase; MDA, malonaldehyde; T-AOC, total antioxidative capabilities. *P < 0.05 vs. the sham group;^#^*P < 0.05 vs. the I/R control group.*

### CH_4_ Inhalation Decreased Apoptosis in Lung Tissue

**FIGURE 6:**
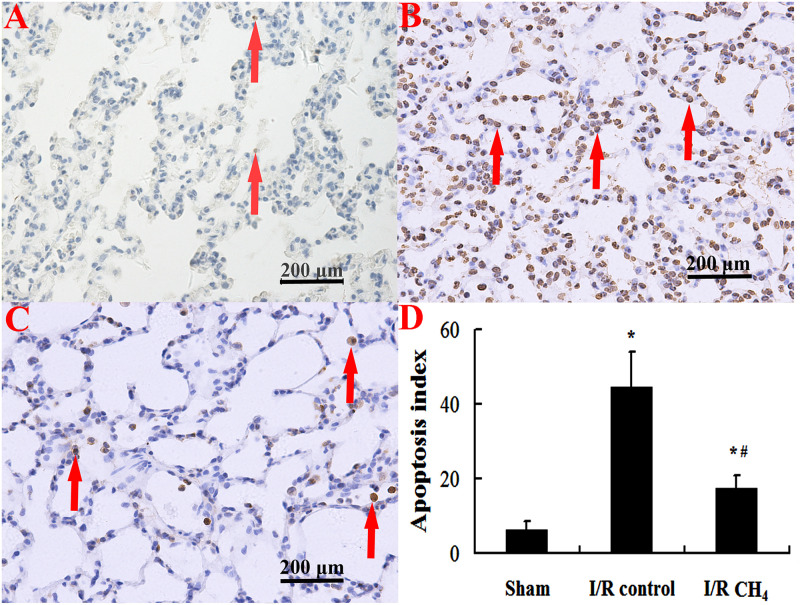
Cellular apoptosis was measured by TUNEL staining (original magnification, 40×). Brown nuclear-stained cells are positive cells (as shown with arrows). There are little positive cells in the sham group, but the number of positive cells in the I/R control group is higher. After CH_4_ treatment, the number of positive cells decreases. TUNEL: terminal deoxynucleotidyl transferase dUTP nick end-labeling; **(A)** sham group; **(B)** I/R control group; **(C)** I/R CH_4_ group; **(D)** apoptosis index (*n* = 4). *P < 0.05 vs. the sham group; ^#^*P* < 0.05 vs. the I/R control group.

**FIGURE 7:**
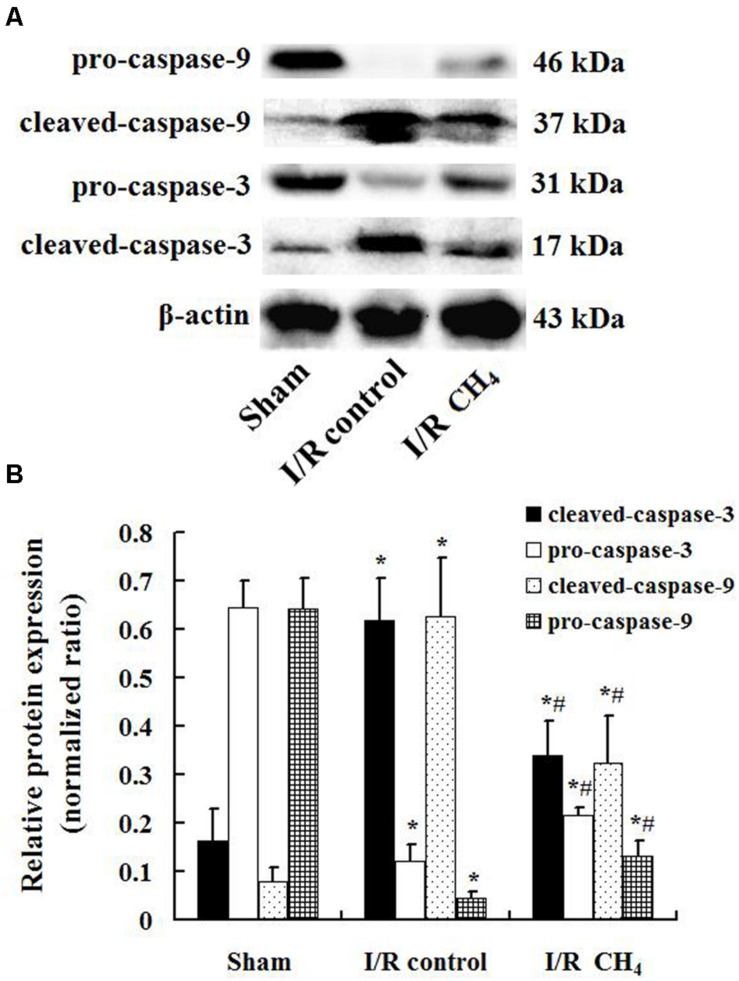
Expression of caspase-3 and caspase-9 by Western blotting (*n* = 4). The expressions of pro-caspase-3, pro-caspase-9, cleaved- caspase-3, and cleaved-caspase-9 in the lung grafts were measured by Western blotting after 180 min of reperfusion in each group. **(A)** Representative bands of pro-caspase-3, pro-caspase-9, cleaved- caspase-3, and cleaved-caspase-9 and β-actin; **(B)** expression of pro-caspase-3, pro-caspase-9, cleaved- caspase-3, and cleaved-caspase-9 normalized to β-actin. Data are shown as mean ± SD. *P < 0.05 vs. the sham group; ^#^*P* < 0.05 vs. the I/R control group.

Compared with that of the sham group, the number of TUNEL-positive cells in the I/R control group increased significantly and that of the I/R CH_4_ group decreased significantly (*P* < 0.05). Therefore, the AI in the I/R control group (44.6 ± 9.4) was significantly higher than that of the sham group (6.4 ± 2.2), and the AI in the I/R CH_4_ group (17.4 ± 3.3) was lower than that of the I/R control group (*P* < 0.05) (Figure 6). The protein expression of cleaved-caspase-3 and cleaved-caspase-9 in the I/R control group were significant higher than that of the sham group, and that of the I/R CH_4_ group decreased significantly (*P* < 0.05). Converse trends were observed in the protein expression of pro-caspase-3 and pro-caspase-9 (Figure 7).

### CH_4_ Inhalation Activated the Nrf2 Pathway

**FIGURE 8:**
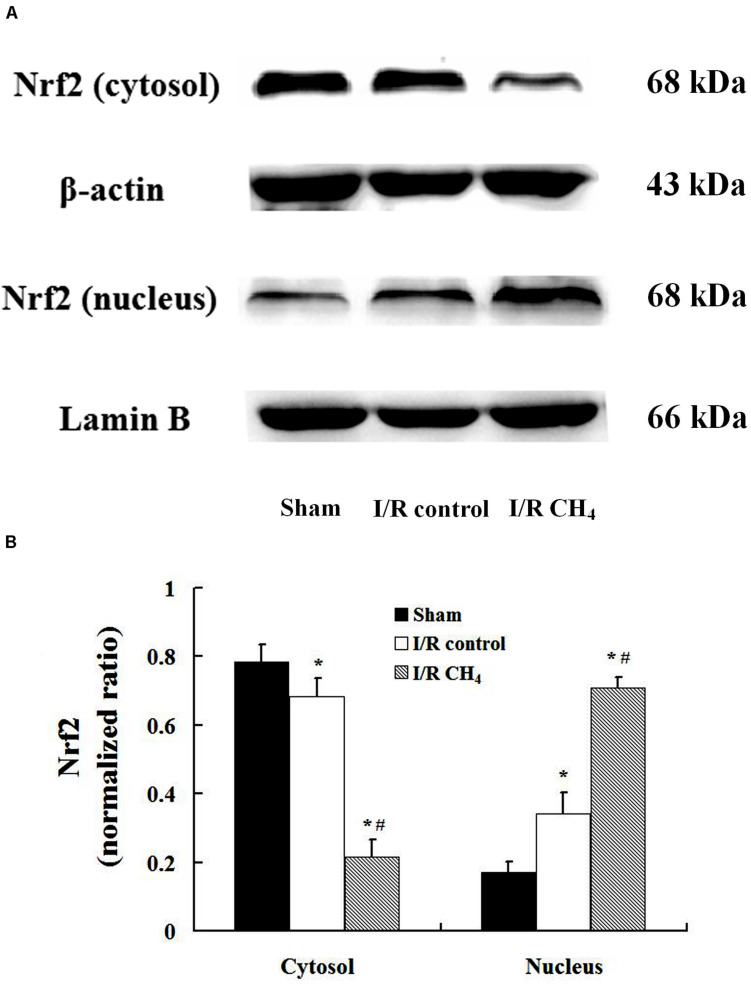
Expression of Nrf2 by Western blotting (*n* = 4). The expression of Nrf2 in the lung grafts was measured by Western blotting after 180 min of reperfusion in each group. **(A)** Representative bands of Nrf2, β-actin, and lamin B; **(B)** expression of Nrf2 normalized to β-actin and lamin B. Data are shown as mean ± SD. Nrf2: nuclear factor E2-related factor. *P < 0.05 vs. the sham group; ^#^*P* < 0.05 vs. the I/R control group.

Compared with that of the sham and I/R control groups, the nucleus Nrf2 protein expression in the I/R CH_4_ group increased significantly (*P* < 0.05). Additionally, Nrf2 protein expression in the cytosol showed a trend that was contrary to that in the nucleus (Figure 8).

## Discussion

CH_4_ is a non-toxic gas that can be produced by bacteria within the human body. CH_4_, similar to nitric oxide, carbon monoxide, and hydrogen sulfide, also plays an important role in the biological systems (Poles et al., 2019). Studies have shown that CH_4_ can penetrate membranes and diffuse into the organelles, including the nucleus and mitochondria (Poles et al., 2019). In 2012, Boros et al. (2012) found that CH_4_ inhalation decreased intestinal I/R injury. More recently, CH_4_ was shown to exert protective effects in the liver, spinal, and lung I/R injury (Ye et al., 2015; Wang et al., 2017, 2020). These results suggest that CH_4_ may be a good therapeutic agent.

In this study, CH_4_ inhalation decreased MPO activity, the levels of IL-1β and TNF-α, MDA activity, NF-κB protein expression, and cell apoptosis, increased IL-10 levels and SOD and T-AOC activities, maintained lung tissue structure and pulmonary surfactant, and ameliorated oxygenation function and lung compliance in rats with lung I/R injury. Additionally, CH_4_ inhalation also up-regulated the protein expression of Nrf2.

Pulmonary surfactant is synthesized in the endoplasmic reticulum of type II alveolar epithelial cells. Phospholipids play a major role in reducing the alveolar gas-liquid surface tension. The surfactant proteins can be divided into active LAs and inactive SAs according to density, including SP-A, SP-B, and SP-C, which are mainly LAs, and SP-D, which is mainly in SAs. A previous study indicated that LAs were the metabolic precursors of SAs (Baritussio et al., 1984), and the composition and metabolism of pulmonary surfactant were recognized as important factors in lung I/R injury (Novick et al., 1996). In this study, CH_4_ inhalation inhibited the decrease in phospholipid level, LA level, and LA percentage induced by I/R, slowed the conversion of LA to SA, and improved pulmonary surfactant, lung compliance, lung oxygenation function, and lung tissue structure. This finding also demonstrated that CH_4_ had practical effects on improving pulmonary surfactant. In 2002, Maitra et al. (2002) found that the rapid conversion of LA to SA occurred in acute lung injury induced by hyperventilation and lung I/R injury induced by transplantation in dogs. In 2004, Meng et al. (2004) demonstrated that exogenous pulmonary surfactant protected against lung I/R injury in isolated rat lungs and maintained the lung tissue structure. In 2019, decreased levels of phosphatidylcholine and total phospholipids were found in lung injury induced by intestinal I/R in rats (Zhao et al., 2019). These results were similar to those of our study. However, the mechanisms by which CH_4_ protests of pulmonary surfactant are still unclear.

Inflammation is recognized as a crucial component of lung I/R injury (den Hengst et al., 2010). It has been proven that CH_4_ can alter the inflammatory balance during inflammatory pathology (Boros et al., 2015). TNF-α is a powerful inflammatory mediator that can increase the permeability of alveolar capillaries and exacerbate lung edema (Bai et al., 2013). IL-1β is also a potential mediator that induces the inflammatory response and has similar effects as those of TNF-α (Wu et al., 2013). IL-10 is an anti-inflammatory mediator that inhibits neutrophil activation and the release of pro-inflammatory cytokines (Rojas et al., 2017). In this study, CH_4_ inhalation decreased the inflammatory response induced by I/R, as demonstrated by the decreased NF-κB, TNF-α, IL-1β, and MPO levels, and the number of total cells and neutrophils in BALF, increased levels of IL-10, and attenuated lung edema. Many studies had similar results. In models of hepatic I/R injury, spinal cord I/R injury, and myocardial ischemia injury in rats, CH_4_ exerted powerful anti-inflammatory effects (Ye et al., 2015; Chen et al., 2016; Wang et al., 2017). Additionally, in lung tissue, the loss of pulmonary surfactant leads to exacerbation of lipopolysaccharide (LPS)-mediated lung inflammation (Arroyo et al., 2019). In 2010, Goto et al. (2010) showed that SP-A decreased the inflammatory response induced by bleomycin-induced acute lung injury via inhibiting inflammatory cytokines, such as TNF-α and IL-1β. Ingenito et al. (2001) showed that the down-regulation of SP-B led to pulmonary dysfunction in a murine model of acute lung injury by regulating inflammatory cytokines, such as TNF-α and IL-1β. In 2020, Yang et al. (2020) showed that inflammatory cytokines such as IL-8 also inhibited SP-A and SP-B and eventually led to lung injury in LPS-induced normal A549 cells. Therefore, CH_4_ may affect the inflammatory response in lung I/R injury by regulating pulmonary surfactant.

Oxidative stress injury is the initial mechanism of I/R injury (Lalkovičová and Danielisová, 2016). MDA is one of the most important products of membrane lipid peroxidation, and the degree of membrane system damage can be estimated by the MDA level. SOD is an anti-oxidative metal enzyme that plays an important role in the balance of oxidation and anti-oxidation. T-AOC is an indicator of the total antioxidant level. In this study, the anti-oxidative indices, including SOD and T-AOC, increased under CH_4_ treatment, and the oxidative index MDA decreased. The antioxidant effect of CH_4_ has been demonstrated in many studies. Liu et al. (2016) reported that CH_4_ attenuated retinal I/R injury by reducing the level of MDA and increasing the level of SOD. The anti-oxidative effect of CH_4_ was also observed in the hepatic I/R injury, spinal cord I/R injury, and myocardial ischemia injury, as shown by the decreased MDA and increased SOD (Ye et al., 2015; Chen et al., 2016; Wang et al., 2017). In addition, pulmonary surfactant could also influence the oxidative stress injury. Mathew and Sarada (2018) reported that the composition of pulmonary surfactant changed and returned to homeostatic levels in hypoxic A549 cells, which was related to reinforcing the induction of phase II antioxidant enzymes. Bezerra et al. (2019) found that exogenous pulmonary surfactant attenuated hyperoxia-induced mouse lung injury by decreasing MDA and increasing SOD. Therefore, the anti-oxidative effects of CH_4_ on lung I/R injury may be related to the regulation of pulmonary surfactant.

Eventually, the accumulation of inflammatory injury, DNA oxidation, and lipid peroxidation leads to cell apoptosis or death (Aslan et al., 2008). Caspase-9 is an initiator caspase that can be stimulated by many factors and causes a caspase cascade. Caspase-3 is a kind of executioner caspase. The cleaved-caspase-3 and cleaved-caspase-9 are the activated forms. In this study, CH_4_ inhalation decreased the number of TUNEL-positive cells and the protein expression of cleaved-caspase-3 and cleaved-caspase-9. Thus, CH_4_ showed an anti-apoptotic effect in this study, which has also been observed in previous studies. Song et al. (2015) found that CH_4_ decreased the number of TUNEL-positive cells and caspase-3 activity in I/R injury in abdominal skin flaps in rats. In the study by Chen et al. (2016), CH_4_ inhibited the increase in TUNEL-positive cells and caspase-3 and caspase-9 protein expression induced by myocardial ischemia. The cleaved-caspase-3 and cleaved-caspase-9 activities were decreased by CH_4_ treatment in spinal cord I/R injury model (Wang et al., 2017). In addition, a strong correlation exists between pulmonary surfactant and apoptosis. Goto et al. (2010) found that TUNEL-positive cells and caspase-3 activity increased a model of bleomycin-induced acute lung injury in SP-A^–/–^ mice, and exogenous SP-A inhibited these effects. Yang et al. (2020) showed that silencing SP-A and SP-B resulted in apoptosis by increasing caspase-3 and caspase-9 protein expression in LPS-induced A549 cells. Therefore, CH_4_-mediated inhibition of apoptosis may occur directly through changes of pulmonary surfactant or indirectly through the anti-inflammatory response and anti-oxidative effects.

Presently, the mechanism of CH_4_ is still unclear. Zhang et al. (2017) found that CH_4_ protected against cerebral I/R injury in rats via a PI3K/Akt/HO-1-dependent anti-oxidative pathway. CH_4_ was also shown to protect against liver injury induced by carbon tetrachloride in mice via PI3K/Akt/GSK-3β-mediated anti-inflammatory effects (Yao et al., 2017). Wang et al. (2019) showed that the NF-κB and MAPK pathways are also involved in the protective effects of CH_4_ in a mouse model of acetic acid-induced ulcerative colitis. In this study, CH_4_ inhalation activated the Nrf2 pathway and protected against lung I/R injury. Wang et al. (2017) also showed that the Nrf2 pathway mediated CH_4_-induced protective effects on spinal cord I/R injury in rats. A previous study reported that Nrf2 not only triggered antioxidants and maintained the balance of lung oxidants and antioxidants, but also altered the production of pulmonary surfactant proteins and maintained surfactant homeostasis (Mathew and Sarada, 2018), which was similar to the results of this study. Therefore, CH_4_ inhalation could protect against lung I/R injury via Nrf2-mediated protection of pulmonary surfactant.

In 2020, Wang et al. (2020) reported that intraperitoneal injection of methane-rich saline decreased lung I/R injury in rats and proved the importance of the PI3K-AKT-NFκB signaling pathway in the protective effects of CH_4_. Compared with those of Wang’ study (Wang et al., 2020), a different method of CH_4_ administration, inhalation, was chosen in this study. Although methane-rich saline is considered safer, inhalation is a more convenient route of administration and acts on the target organ (lung) directly. CH_4_ is flammable in a narrow range of concentrations (5–15%) in air (Liu et al., 2012); thus, controlling methane concentration and careful monitoring can completely avoid the risk of explosion. Therefore, the method of CH_4_ inhalation is very important and cannot be ignored. Additionally, in this study, we further observed the effect of CH_4_ inhalation on pulmonary surfactant, and the results suggest that CH_4_ may alleviate lung I/R injury through Nrf2-mediated pulmonary surfactant protection.

This study also has some limitations. First, only 3 h following reperfusion was observed, and a longer period of time may be required to observe the long-term effects of CH_4_ inhalation on lung I/R injury. Second, only one CH_4_ concentration was used in this study. Different CH_4_ concentrations may show different effects, and the optimal CH_4_ concentration should be evaluated. Third, different components of pulmonary SP-A, SP-B, SP-C, and SP-D, should be measured so that the effect of CH_4_ on pulmonary surfactant would be more specific. Then, the Nrf2 is knocked down/out genetically or by using specific pharmacological inhibitor would further verify the causative role of Nrf2 and this will be studies in the future research. Finally, the CH_4_ concentration in blood was not monitored during inhalation. However, the rats did not have any adverse reactions during the experiment. Previous studies also demonstrated that CH_4_ inhalation had no adverse reactions in pigs and rats, and CH_4_ in the blood showed rapid metabolism (Mészáros et al., 2017; Bari et al., 2019).

## Conclusion

Our results demonstrated that CH_4_ inhalation can exert protective effects on lung I/R injury in rats by decreasing the inflammatory response, inhibiting oxidative stress injury, and attenuating cell apoptosis, which is related to Nrf2-mediated pulmonary surfactant protection. Due to its non-toxic characteristics and membrane permeability, CH_4_ will be a new therapeutic gas.

## Data Availability Statement

The raw data supporting the conclusions of this article will be made available by the authors, without undue reservation, to any qualified researcher.

## Ethics Statement

The animal study was reviewed and approved by the Ethics Committee of the Affiliated Hospital of Qingdao University.

## Author Contributions

BZ, XT, and CM designed the experiments. BZ, XT, GL, and HZ performed the experiments. CM and XW analyzed the data. YY and JY contributed to the reagents. BZ and CM wrote the manuscript. All authors contributed to the article and approved the submitted version.

## Conflict of Interest

The authors declare that the research was conducted in the absence of any commercial or financial relationships that could be construed as a potential conflict of interest.
